# Outcome after Desensitization in HLA or ABO-Incompatible Kidney Transplant Recipients: A Single Center Experience

**DOI:** 10.1371/journal.pone.0146075

**Published:** 2016-01-05

**Authors:** Teresa Kauke, Sandra Klimaschewski, Ulf Schoenermarck, Michael Fischereder, Andrea Dick, Markus Guba, Manfred Stangl, Jens Werner, Bruno Meiser, Antje Habicht

**Affiliations:** 1 Laboratory for Immunogenetics, University Hospital LMU, Munich, Germany; 2 Clinic for General, Visceral-, Transplantation-, Vascular- and Thoracic Surgery, University Hospital LMU, Munich, Germany; 3 Transplant Center, University Hospital LMU, Munich, Germany; 4 Department of Internal Medicine IV, Renal Division, University Hospital LMU, Munich, Germany; Universidade de Sao Paulo, BRAZIL

## Abstract

**Background:**

The shortage of deceased donors led to an increase of living donor kidney (LDK) transplantations performed in the presence of donor-specific antibodies (DSA) or ABO incompatibility (ABOi) using various desensitization protocols.

**Methods:**

We herein analyzed 26 ABOi and 8 Luminex positive DSA patients who were successfully desensitized by anti-CD20, antigen-specific immunoadsorption and/or plasmapheresis to receive an LDK transplant. Twenty LDK recipients with non-donor-specific HLA-antibodies (low risk) and 32 without anti-HLA antibodies (no risk) served as control groups.

**Results:**

1-year graft survival rate and renal function was similar in all 4 groups (creatinine: 1.63 ± 0.5 vs 1.78 ± 0.6 vs 1.64 ± 0.5 vs 1.6 ± 0.3 mg/dl in ABOi, DSA, low risk and no risk group). The incidence of acute T-cell mediated rejections did not differ between the 4 groups (15% vs 12, 5% vs 15% vs 22% in ABOi, DSA, low risk and no risk), while antibody-mediated rejections were only found in the DSA (25%) and ABOi (7.5%) groups. Incidence of BK nephropathy (BKVN) was significantly more frequent after desensitization as compared to controls (5/34 vs 0/52, p = 0.03).

**Conclusion:**

We demonstrate favorable short-term allograft outcome in LDK transplant recipients after desensitization. However, the desensitization was associated with an increased risk of BKVN.

## Introduction

The considerable shortage of organ donors and increasing number of patients with end-stage renal disease has led to an extended waiting time for potential renal allograft recipients. In addition, approximately 30% of patients on the kidney waiting list are sensitized to HLA antigens of potential donors facing a significant increased waiting time [1]. The introduction of solid phase assays, such as the Luminex technology, which allows a more sensitive and specific identification of donor-specific anti-HLA antibodies (DSA), may impact the organ allocation process even further, in that even more patients are considered to be sensitized and rejected as recipients [2;3]. However, the impact of DSA detected only by solid phase assays on transplant outcome remains under debate [4]. While the identification of DSA in the presence of a negative complement depending cytotoxicity (CDC) in pre-transplant sera were linked with increased immunogenic graft loss in retrospective analysis [5;6], smaller prospective series questioned their relevance [7;8]. In particular, the degree of mean fluorescent intensity (MFI) that is of clinical relevance remains unclear, with large retrospective series showing no apparent difference in graft loss in recipients with DSA at an MFI < 3000 [9]. Therefore, several centers developed desensitization protocols to allow immunized patient to receive a kidney from a donor across the HLA barrier. Using different therapeutic strategies with new immunosuppressive medications, modern apheresis techniques and/or antigen-specific immunoadsorption, promising short-term outcomes have been reported [10–12]. Furthermore, ABO-incompatible (ABOi) living kidney donor (LDK) transplantation has become a popular alternative to expand the donor pool [13].

However data from those studies have been difficult to interpret or compare because of heterogenecity among immunologic testing techniques, DSA levels, desensitization strategies, and demographic and clinical characteristics of donor and recipient populations. We therefore aimed to reevaluate our own practice by assessing 1-year graft outcome after desensitization in renal transplant recipients who received a kidney from a living donor across blood group and/or HLA barriers in comparison to two matched control groups with low immunological risk. We furthermore aimed to evaluate the impact of the intensified immunosuppressive regiment on the rate of BK virus infections.

## Methods

### Study design and patients

We conduct a retrospective cohort study with 91 adult patients (> 18 years) who received a LDK between January 2007 and June 2012 at our center. The study was approved by the ethics board of the Ludwig-Maximilians-University Munich. The patients gave their written consent. This consent procedure was also approved by the ethics committee of the Ludwig-Maximilians-University Munich (Approval number 513–12). The patients selected for the study had either Luminex-detected donor-specific antibodies with a MFI > 3000 or DSA with a MFI < 3000 but a positive CDC B-cell and/or Luminex crossmatch prior to transplantation (DSA group, n = 8) or had received a kidney from an ABO blood group incompatible donor (ABOi group; n = 26). The patients selected for the control groups were recipients, who received a living-donor kidney during the same time period and were maintained on the same long-term tripple immunosuppressive regiment and had Luminex-detected non-donor-specific antibodies (nDSA) (low risk group, n = 20) or no anti HLA-antibodies (no risk group, n = 32). All patients had a negative T-cell CDC crossmatch. However, within the DSA group 4/8 patients had a positive B-cell CDC crossmatch and 6/8 patients had a positive Luminex crossmatch before desensitization.

### HLA-antibody screening and C1q assay

Recipients were screened for the presence of HLA-antibodies before transplantation and during the routine follow-up by means of Luminex (Life Screen Deluxe, Gen-Probe, USA). In positively screened patients antibody specificity in relation to the donor was confirmed by Single Antigen Beads (SAB) (LSA, Gen-Probe, USA). An MFI of > 3000 was used as cut-off. Recipients in the DSA group were screened for C1q binding HLA-antibodies by C1q-SAB assay using Luminex (LabScreen, One lambda, USA).

### Isoagglutinine titers

Anti-donor isoagglutinin titers were measured by a microtube column agglutination technique, using the Diamed-Coombs-Anti-IgG^®^ and Diamed-ID-NaCl^®^ systems (DiaMed Diagnostika Deutschland, Germany).

### Immunosuppressive protocol

Maintenance immunosuppression in all patients consisted of a triple drug therapy including either tacrolimus (trough levels 8–12 ng/mL during the first 3 month, 6–10 ng/ml during month 3–6, 4–8 ng/ml during month 6–12 post-transplantation) or ciclosporin (trough levels 160–200 ng/mL during the first 3 month, 120–160 ng/ml during month 3–6, 80–120 ng/ml during month 6–12 post-transplantation), mycophenolatemofetil (1000 mg orally twice daily) and methylprednisolone (tapered to the dose of 5 mg by 6 month post-transplantion). Patients received desensitization and/or induction therapy according to the following protocol (Table 1):

**Table 1:** Protocols for desensitization and immunosuppressiv therapy.

Group	Rituximab	Desensitization	Induction Therapy	Maintenance Therapy
DSA	+	Plasmaexchange	ATG	+
ABOi	+	Antigen-specific IA	ATG	+
low-risk	-	-	ATG/Basiliximab	+
no risk	-	-	-	+

Rituximab (MabThera®, Roche Pharmaceuticals, Basel, Switzerland): 375 mg/m^2^, 4 weeks prior to transplantation; ATG Fresenius® (Fresenius, Munich, Germany): 4 mg/kg, once a day, day 0–4 post transplantation; Basiliximab (Simulect^®^, Novartis, Basel, Switzerland): 20 mg day 0 and day 4 post transplantation, Antigen-specific Immunoadsorption (Glycosorb A/B^@^ columns, GlycorexTransplantation AB, Lund, Sweden)

### Anti-infective prophylaxis

All patients with a high-risk CMV constellation (D +/R -) received a prophylaxis with valganciclovir for 3 months post-transplantion. Furthermore, oral PcP prophylaxis (trimethoprim/sulfamethoxazole 160 mg/800 mg, 3 times a week) was administered 6 months post-transplant to every patient.

### Antigen-specific immunoadsorption and plasma exchange

Immunoadsorption (IA) was performed using a commercially available apheresis device (Octo Nova^®^, Diamed Medizintechnik, Germany) and hollow-fibre plasma separators (Plasmaflow^®^ OP-05, Asahi Kasei Medical Ca. LTD, Tokyo, Japan). Plasma was passed through the antigen-specific carbohydrate column (Glycosorb A/B^®^, Glycorex Transplantation AB, Sweden). Preoperatively ABOi recipients underwent IA every other day until IgG anti-A/B titers were 1:8 or less. Postoperative IA was only performed if anti-A/B IgG titers exceeded 1:8 in the first and 1:16 in the second postoperative week. Plasma exchange (PE) was performed using the same commercially available apheresis device and hollow-fibre plasma separators. Each patient within the DSA group was cleared for transplantation after receiving standard 6 PE. At each session one-plasma volume was replaced by 5% serum albumin.

### BKV

Systematic BKV screening using a polymerase-chain-reaction (PCR) assay was routinely performed at baseline (transplantation) and every three months thereafter. The cut off used to considerer the PCR as a positive viremia for BK virus was 1,000 copies/ml. In patients with diagnosed BK viremia and/or BK nephropathy (BKVN) immunosuppression was reduced, in that MMF was slowly tapered to half the dose and blood through levels of tacrolimus were reduced (goal 4–5 ng/ml). In the event of BKVN with concomitant acute rejection MMF was replaced by leflunomide. Leflunomide was administered with a loading dose of 100 mg for 3 days, followed by maintenance dose of 20 mg/d, adjusted for blood levels of 50–100 μg/ml.

### Biopsies/Rejections

Allograft biopsies were taken when renal graft function was impaired or BK viremia developed. Rejection was determined according to the diagnostic criteria proposed at the 2007 Banff Conference [14]. C4d staining was routinely performed in paraffin sections of all biopsies. C4d positive staining in peritubular capillaries (PTC) was evaluated semi-quantitatively as follows: minimal (<10% of PTC), focal (11–50% of PTC) and diffuse (>50% of PTC) [15]. The diagnosis of BK nephropathy was based on histologic features in combination with positive staining for SV40.

### Demographic and clinical information

The following parameters were evaluated:

1. Donor variables: age, gender, relationship to recipient, CMV and EBV status
2. Recipient characteristics: age, gender, cause of kidney failure, time on dialysis, previous transplants, human leukocyte antigen (HLA) mismatch, panel reactive antibodies (>10%), anti-HLA-antibodies, CMV- and EBV-status, bodymass index (BMI) [kg/m^2^], anti-donor isoagglutinine IgG titers, number of performed PE or IA therapies.
3. Perioperative factors: cold ischemia time
4. Post-transplant factors: serum creatinine levels and calculated glomerular filtration rate (GFR) by MDRD; proteinuria, defined as > 500 (mg)protein/(g)creatinine ratio in spot urine; loss of GFR within the first year after transplantation (GFR_at 1 month_—GFR_at 12 month_), number of rejections (confirmed in all cases by graft biopsy) and number of BK-virus infections or nephropathy (serological or biopsy proven) within 12 month

### Statistics

Statistical analysis was performed using Sigma Plot. Data are given as mean values ± standard deviation and n represents the number of patients per group. To calculate differences between group we utilized the Mann-Whitney-U-test for continuous variables and ChiSquare-test for categorical variables. A value of probability of less than 0.05 was defined to indicate statistical significance.

## Results

### Patient characteristics

The patient characteristics are shown in Table 2. No significant differences were noted in the age and sex of donors and recipients, relationship between them, kidney diseases and the number of HLA mismatches. As expected the number of prior transplants was significantly higher in the DSA group (75%) and low risk group (40%) resulting in significantly higher PRA (panel reactive antibodies) IgG levels as in the no risk and ABOi group. Interestingly, time on dialysis was also higher in the DSA group.

**Table 2:** Patient and donor characteristics.

	AB0i	DSA	low Risk	no Risk	p-value
Number (n)	26	8	20	32	
Age recipient (years)	49.5 ± 15.3	45 ± 5.6	44.3 ± 17	46 ± 13	ns
Female recipient (%)	38.5	37.5	30	47	ns
BMI recipient (kg/m^2^)	24.1 ± 4.3	24.9 ± 5.1	23.1 ± 3.9	25 ± 4	ns
**Kidney Disease**					ns
Diabetes	2	1	0	1	
Hypertension	4	0	2	4	
Glomerulonephritis	8	7	14	20	
ADPKD	4	0	2	2	
Amyloidosis	2	0	0	0	
Genetic	1	0	1	1	
Unknown	5	0	1	5	
Time on dialysis (days)	587 ± 529	836 ± 635	499 ± 381	512 ± 210	ns
Preemptive Tx (%)	27	0	5	31	ns
HLA Mismatch (n)	3.5 ± 1.6	3.0 ± 1.5	3.1 ± 1.6	3.1 ± 1.4	ns
Re-Transplantation (%)	15	75	40	3	< 0.0001
PRA IgG > 10% (%)	11	100	60	0	< 0.0001
Age donor (years)	57.7 ± 10.5	53.1 ± 9.5	54.6 ± 10.7	57 ± 11	ns
Female donor (%)	69	37.5	80	62.5	ns
Cold ischemia time (hours)	1 ± 0.0	1 ± 0.0	1 ± 0.1	1 ± 0.0	ns

All values represent means ± SD, unless otherwise stated. Abbreviations: HLA: human leukocyte antigen; PRA: panel reactive antibody; BMI: body mass index.

### Desensitization within the ABOi and DSA group

The median anti-donor IgG isoagglutinine titer in the ABOi group was 1: 512 (range 1:2 up to 1:1024) prior of treatment. The 26 patients underwent a mean of 4 ± 3 IA sessions before surgery. Five of 26 patients (19.2%) patients required an IA session post-transplantion because of a rise in isoagglutinine titers. All 5 patients had a higher titer before transplantation.

Within the DSA group 1 patient was desensitized due to DSA against HLA-class I only, 1 patient due to DSA against HLA-class I and II, while the remaining 6 patients were positive for DSA against HLA-class II only. The mean DSA against HLA-class I MFI was 1000 ± 1927 and the mean DSA against HLA-class II MFI was 9100 ± 7088 before desensitization. With 6 routine PE treatments the mean DSA against HLA-class I MFI could be reduced to 200 ± 385 while the mean DSA against HLA-class II MFI remained significantly elevated by 6800 ± 7484. Four patients were transplanted with DSA against HLA-class II MFI above 3000.

### Patient and graft survival

Patient survival at 12 months follow-up was 100% in all 4 groups, while graft survival was 100% in the ABOi, DSA and no risk and 95% in the low risk group. 1 patient in the low risk group lost his graft within 1 week after transplantation because of recurrence of disease (FSGS).

### Graft function

**Fig 1:**
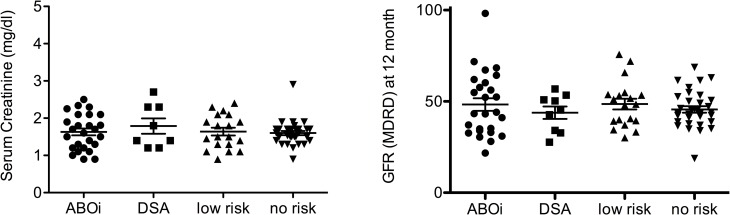
Renal function at 12 month. Renal function, as assessed by serum creatinine levels and estimated GFR (MDRD formula) at 12 months after transplantation.

Renal function, as assessed by serum creatinine levels and estimated GFR (MDRD formula) at 10 days, 1, 4, and 6 months (data not shown) was similar in all 4 groups, while it was slightly reduced in the DSA group at 12 months after transplantation (creatinine: 1.78 ± 0.6 mg/dl; GFR: 42.6 ± 10 ml/min) as compared to the ABOi, low risk and no risk group (creatinine: 1.63 ± 0.5 vs 1.64 ± 0.5 vs 1.6 ± 0.3 mg/dl; GFR: 48.05 ± 18 vs 48.5 ± 13 vs 45.4 ± 10 ml/min, respectively). (Fig 1) However, the difference was not statistically significant. While no patient in the no risk group and ABOi group developed significant proteinuria defined as > 500 mg protein/ g creatinine ratio, 1 DSA (2950 mg protein/g creatinine) and 2 low risk patients (530 mg protein/g creatinine and 1010 mg protein/g creatinine, respectively) showed significant proteinuria (p = 0.935).

### Loss of graft function within 12 months

**Fig 2:**
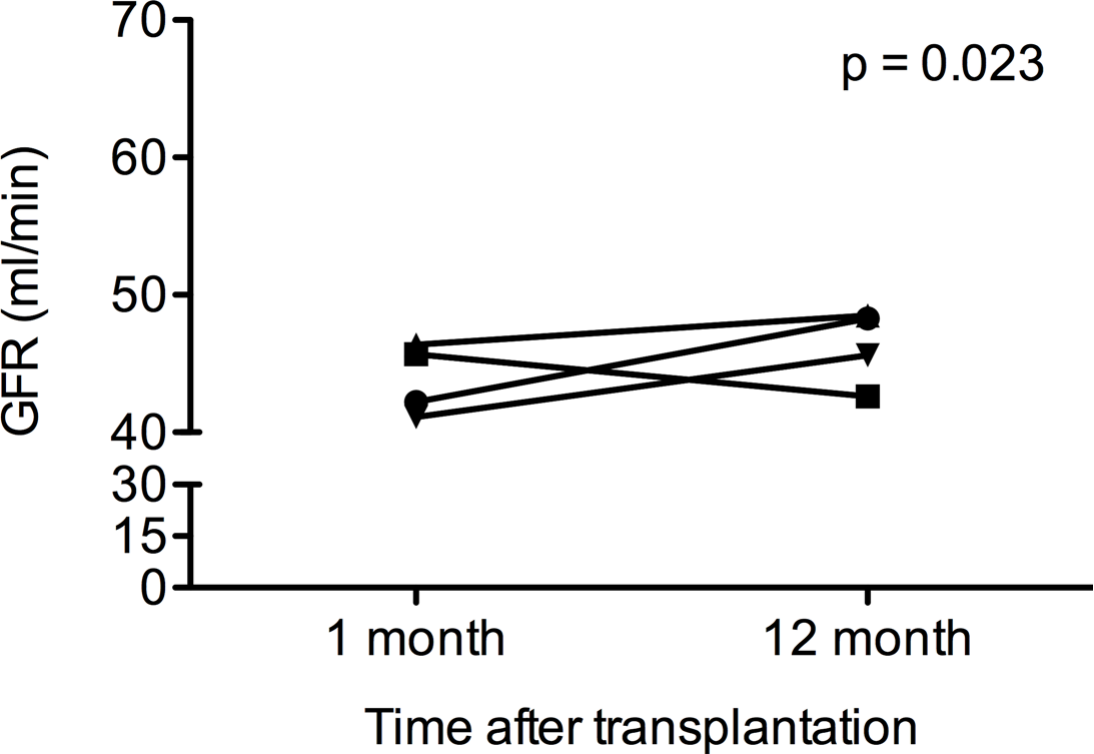
Loss of renal function within the first year. The GFR slope within the first year was assessed according to the following formula: GFR _at 1 month_−GFR _at 12 month_.

To estimate loss of renal function within the first year after transplantation we evaluated the GFR slope (GFR _at 1 month_−GFR _at 12 month_). Renal function increased in the AB0i (GFR difference: + 6.1 ± 11,8 ml/min), low risk (GFR difference: + 2,1 ±12,5 ml/min) and no risk group (GFR difference: + 4,5 ± 12,5 ml/min), while it significantly decreased in the DSA group (GFR difference: -3,1 ± 7,5 ml/min, p = 0.023). (Fig 2)

### Rejections

**Fig 3:**
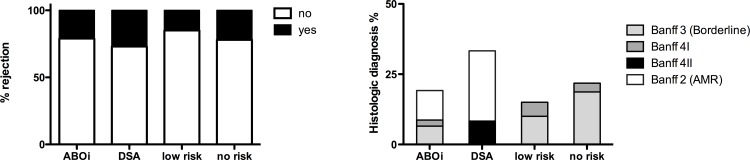
Incidence of rejection. The number of patient experiencing 1 or more biopsy proven rejections (including borderline cases with impaired renal graft function) according to the Banff 97 classification: 2 = antibody mediated rejection (AMR), 3 = borderline lesion, 4 I and 4 II = acute cellular rejection.

Fig 3 illustrates that the number of patients experiencing 1 or more rejections (including borderline cases) was higher in the DSA group (37.5%) in comparison to the ABOi (23%), low risk (15%) and no risk group (22%). However, the difference did not reach statistical significance (p = 0.641). While the number of T-cell mediated rejections was comparable in all 4 groups (15% vs 12,5% vs 15% vs 22% in ABOi, DSA, low risk and no risk group, p = 0.868) antibody-mediated rejection (AMR) only occurred in 2 DSA (25%) and 2 ABOi patients (8%). Of note, 2 additional ABOi recipients developed a T cell mediated rejection after reduction of immunosuppression due to BKVN.

### HLA antibody screening post-transplant

#### Persistence of pre-existing DSA

Pre-existing DSA against HLA-class II levels with a MFI value above 3000 were still detectable in 6 (75%) patients including those 3 who experienced an acute rejection episode. In 50% of patients the antibodies were able to fix complement C1q. However, C1q-DSA did not seem to predict an acute rejection. (Table 3)

**Table 3:** Immunologic characteristics in the DSA group.

Pat	DSA	C1q	B celXM	LuminexXM	MFI 1	MFI 2	persistentDSA	Rejection	Creatinine	Proteinuria
1	DQ2	No	Negative	Negative	5.000	2.000	Yes	4IIb)	1,4	No
2	DR15	No	Negative	Positive	9.000	200	Yes	No	1,2	No
3	A2	No	Positive	Positive	5.000	600	No	No	1,4	No
4	DQ5	Yes	Negative	Negative	20.000	17.000	Yes	No	2,3	No
5	B7 &DR16	Yes	Positive	Positive	9.000	6.000	Yes	2	2,7	Yes
6	DQ6	No	Positive	Positive	1.800	200	No	No	1,4	No
7	DR13	Yes	Negative	Positive	18.000	17.000	Yes	2	2,3	No
8	DR3	Yes	Positive	Positive	12.000	10.000	Yes	No	1,8	No

DSA: donor specific anti-HLA antibody, XM: crossmatch, Tx: Transplantation, MFI: Mean Fluorescence Intensity, MFI 1: before PE, MFI 2: at Tx; PE: Plasma Exchange, Rejection: according to Banff classification; Banff: Banff classification, 4IIb: cellular, 2: humoral;. Proteinuria: > 500 mg protein/g creatinine at 12 month post tx, creatinine mg/dl at 12 month post tx, C1q: C1q binding donor-specific anti-HLA antibodies,

#### Persistence of pre-existing nDSA

In the low risk group we found evidence of persistent nDSA in 5/17 patients. All of these patients were biopsied due to impaired renal function. However, none of them experienced an acute rejection.

#### Development of denovo DSA

Eight patients developed denovo DSA during follow up (2/24 ABOi, 3/17 low-risk and 3/29 no-risk patients). Only 1 ABOi recipient showed histologic evidence of AMR.

#### Development of denovo nDSA

Denovo nDSA could be detected in 2 ABOi (8,4%), 2 low risk (11,8%) and 4 no risk patients (13,7%). 1 ABOi recipient developed a Banff 3 borderline lesion, 1 low risk patient developed a Banff 4 Ia rejection and 1 no risk patient developed a Banff 3 borderline lesion.

In summary, the persistence of DSA was associated with a frequency of 50% (3/6 patients) acute rejection episodes, in comparison to 37,5% in recipients with denovo nDSA (3/8 patients) and 12.5% (1/8 patients) in recipients with denovo DSA.

### BKV

Overall 6/86 patients (6,9%) developed a significant BKV (median 165500 copies/ml) at a median of 192 ± 65 days after transplantation leading to BKVN in 5/86 patients (incidence of 5.8%). All patients with BK-viremia/BKVN were within the ABOi (4/26) and DSA (2/8) group while no patient in the no risk or low risk group showed BK- viremia (0/52) (p = 0,03). BK-viremia/BKVN developed in 2/6 patients (33%) after anti-rejection therapy consisting of steroids, plasmapheresis, IVIG and Rituximab. In patients with diagnosed BK-viremia and/or BKVN immunosuppression was reduced. Two patients experienced biopsy-proven acute rejection thereafter. They were treated by steroid pulse therapy and MMF was replaced by leflunomide. Renal function 12 month after transplantation was reduced in patients with BKVN (creatinine 2,22 ± 0,67 mg/dl) in comparison to those without signs of BKVN (creatinine 1,59 ± 0,44 mg/dl) (Table 4).

**Table 4:** Clinical characteristics in patients with BKV.

Pat.	Group	Rejection prior to BKV	Rejection therapy	BKV(days post tx)	BKV (copies/ml)	BKVN	Intervention to BKVN	Rejection post intervention
1	ABOi	No	No	265	590.000	Yes	Red. IS	No
2	ABOi	No	No	66	430.000	Yes	Red. IS	Yes
3	ABOi	2 (humoral)	Steroids, ATG, PE	192	490.000	Yes	Red. IS	No
4	ABOi	No	No	174	350.000	Yes	Red. IS	Yes
5	DSA	2 (humoral)	Steroids, Rituximab	202	770.000	Yes	Switch	No
6	DSA	2 (humoral)	Steroids, ATG, PE	254	1.500	No	Red. IS	No

Tx: Transplantation, BKVN: BK virus nephropathy, ATG: Anti-thymocyte globulin, PE: Plasma Exchange, Red IS: Reduction in immunosuppression; Switch: switch from MMF to Leflunomid.

## Discussion

In this report, we present our experience in patients that underwent desensitization due to ABO or HLA incompatible living donor kidney transplantation in comparison to two control collectives. Using desensitization with anti-CD20, antigen-specific IA and/or PE we demonstrated favorable short-term allograft survival and function after ABOi renal transplantation. However, in patients with Luminex-detected DSA pre-transplant we noted an increase in rejection episodes and a significant decline in renal function 12 months after transplantation. Furthermore, we noted an increase in the rate of BK virus infections after the intensified desensitization protocol in ABOi and HLA recipients.

Our results are in line with the extended experience of other groups showing that ABO incompatible kidney transplantation is a reasonable way to extend the donor pool. Takahashi K et al recently reported excellent patient (91%) and graft (83%) survival 9 years after kidney transplantation within the largest cohort of 1427 ABO incompatible recipients with the longest follow-up of more than 20 years [16].

In the context of LDK transplant recipients with DSA Klein et al. showed that desensitization results in good outcomes with low rate of side effects [17]. In contrast, Bentall et al., also showed an impaired 5 year graft survival in kidney allograft recipients with a positive pre-transplant flow cytometric crossmatch who were desensitized by PE and IVIG in comparison to a crossmatch-negative control group (70.7% vs. 88.0%) [18]. This is in line with our own results showing a reduced renal function with a loss of GFR of 3.1 ml/min within the first year after transplantation across HLA barriers as compared to the controls. Furthermore, we experienced more rejections in the DSA group with the persistence of DSA beeing the greatest risk to develop a rejection in our cohort. Comparably, Bagnaso et al experienced an incidence of clinical and subclinical rejections of 66% in serial graft biopsies in HLA-incompatible kidney allograft recipients with pre-transplant DSA in the first year [19]. Furthermore, patients with DSA post-transplantation had a significant higher probability to develop transplant glomerulopathy. However, we were unable to show an association of rejection episodes with high MFI DSA titers, C1q-binding DSA and/or a positive Luminex and/or B-cell crossmatch, although this might be an effect of our small sample size. In addition, C1q-binding capacity and the MFI DSA titer did not correlate with CDC crossmatch, as patient number 6 had a very low anti-DQ titer, which was C1q negative, but a positive CDC B-cell XM. He was desensitized due to the positive CDC- B-cell XM. The positive CDC XM might be caused by non-HLA antibodies. Thus while expanding the donor pool by transplanting over HLA barriers one should consider the higher immunologic risk. On the other hand, it has been demonstrated that the longer waiting time on dialysis in immunized versus non immunized recipients is a major risk factor for a poor patient outcome after kidney transplantation [20]. Furthermore, LDK transplantation after desensitization appears beneficial for the patient, in that survival rates in desensitized LDK transplant recipients were strikingly higher than in dialysis patients waiting for a HLA compatible deceased donor organ despite a diminished graft function (80.6% vs 49.1%) [21].

However, the impact of potential side effects of desensitization such as infections or malignancies is still unknown. The BKV is a member of the polyoma virus family with a seroprevalence between 46% - 94% in the general population [22]. With the renal tubular system being the main target for virus infiltration reactivation of the latent BKV in kidney allograft recipients receiving immunosuppression can cause BK specific interstitial nephritis. It mostly develops within the first year and can result in a 50% rate of graft loss [23–26]. A recent increase in prevalence BKVN (1% - 10%) has been attributed, in part, to the use of more potent immunosuppressive regimens and an increased use of antirejection therapy [27;28]. However, it seems the overall intensity of immunosuppression, rather than one specific drug, leading to an increased risk [29]. In line with this theory, we noted a significant increase in the incidence of BKVN in patients after desensitization in comparison to the control groups. The intensified immunosuppressive therapy due to the desensitization and/or the more frequent antirejection therapy during the first year may have rendered those groups more susceptible for BKV reactivation. However, another reason might be a lower maintenance therapy within the low risk groups. These data are in agreement with recent reports including our own showing an increased incidence of BKVN (20–25%) after desensitization in ABOi or HLA incompatible transplant recipients [30–32].

In conclusion we demonstrate impaired short-term allograft outcome in LDK transplant recipients undergoing desensitization for preformed DSA (HLA-incompatible). However, regarding the increased rejection rate we did not succeed in identifying any predictive serologic parameter. Prospective and multicenter studies are needed to standardized desensitization therapies and to proof the predictive values of MFI cut-off or C1q-binding assays.

## References

[1] Smits JM, Van Houwelingen HC, De MJ, Persijn GG, Claas FH Analysis of the renal transplant waiting list: application of a parametric competing risk method. Transplantation 15-11-1998;66:1146–1153. 9825809 10.1097/00007890-199811150-00006

[2] Picascia A, Infante T, Napoli C: Luminex and antibody detection in kidney transplantation. Clin Exp Nephrol 2012;16:373–381. 10.1007/s10157-012-0635-1 22552384

[3] Roelen DL, Doxiadis II, Claas FH: Detection and clinical relevance of donor specific HLA antibodies: a matter of debate. Transpl Int 2012;25:604–610. 10.1111/j.1432-2277.2012.01491.x 22587521

[4] Lee PC, Ozawa M, Hung CJ, Lin YJ, Chang SS, Chou TC: Reappraisal of HLA antibody analysis and crossmatching in kidney transplantation. Transplant Proc 2009;41:95–98. 10.1016/j.transproceed.2008.10.074 19249487

[5] Patel AM, Pancoska C, Mulgaonkar S, Weng FL: Renal transplantation in patients with pre-transplant donor-specific antibodies and negative flow cytometry crossmatches. Am J Transplant 2007;7:2371–2377. 17845571 10.1111/j.1600-6143.2007.01944.x

[6] Bielmann D, Honger G, Lutz D, Mihatsch MJ, Steiger J, Schaub S: Pretransplant risk assessment in renal allograft recipients using virtual crossmatching. Am J Transplant 2007;7:626–632. 17352712 10.1111/j.1600-6143.2007.01667.x

[7] Phelan D, Mohanakumar T, Ramachandran S, Jendrisak MD: Living donor renal transplantation in the presence of donor-specific human leukocyte antigen antibody detected by solid-phase assay. Hum Immunol 2009;70:584–588. 10.1016/j.humimm.2009.05.007 19477211

[8] van den Berg-Loonen EM, Billen EV, Voorter CE, van Heurn LW, Claas FH, van Hooff JP, Christiaans MH: Clinical relevance of pretransplant donor-directed antibodies detected by single antigen beads in highly sensitized renal transplant patients. Transplantation 27-4-2008;85:1086–1090. 10.1097/TP.0b013e31816b3ed1 18431226

[9] Susal C, Ovens J, Mahmoud K, Dohler B, Scherer S, Ruhenstroth A, Tran TH, Heinold A, Opelz G: No association of kidney graft loss with human leukocyte antigen antibodies detected exclusively by sensitive Luminex single-antigen testing: a Collaborative Transplant Study report. Transplantation 27-4-2011;91:883–887. 10.1097/TP.0b013e3182100f77 21325993

[10] Susal C, Morath C: Current approaches to the management of highly sensitized kidney transplant patients. Tissue Antigens 2011;77:177–186. 10.1111/j.1399-0039.2011.01638.x 21299521

[11] Reinsmoen NL: New approaches for optimizing transplant of sensitized patients. Curr Opin Organ Transplant 2012;17:406–408. 10.1097/MOT.0b013e328355f3c4 22790075

[12] Vo AA, Lukovsky M, Toyoda M, Wang J, Reinsmoen NL, Lai CH, Peng A, Villicana R, Jordan SC: Rituximab and intravenous immune globulin for desensitization during renal transplantation. N Engl J Med 17-7-2008;359:242–251. 10.1056/NEJMoa0707894 18635429

[13] Montgomery JR, Berger JC, Warren DS, James NT, Montgomery RA, Segev DL: Outcomes of ABO-incompatible kidney transplantation in the United States. Transplantation 27-3-2012;93:603–609. 10.1097/TP.0b013e318245b2af 22290268 PMC3299822

[14] Solez K, Colvin RB, Racusen LC, Haas M, Sis B, Mengel M, Halloran PF, Baldwin W, Banfi G, Collins AB, Cosio F, David DS, Drachenberg C, Einecke G, Fogo AB, Gibson IW, Glotz D, Iskandar SS, Kraus E, Lerut E, Mannon RB, Mihatsch M, Nankivell BJ, Nickeleit V, Papadimitriou JC, Randhawa P, Regele H, Renaudin K, Roberts I, Seron D, Smith RN, Valente M: Banff 07 classification of renal allograft pathology: updates and future directions. Am J Transplant 2008;8:753–760. 10.1111/j.1600-6143.2008.02159.x 18294345

[15] Mengel M, Sis B, Haas M, Colvin RB, Halloran PF, Racusen LC, Solez K, Cendales L, Demetris AJ, Drachenberg CB, Farver CF, Rodriguez ER, Wallace WD, Glotz D: Banff 2011 Meeting report: new concepts in antibody-mediated rejection. Am J Transplant 2012;12:563–570. 10.1111/j.1600-6143.2011.03926.x 22300494 PMC3728651

[16] Takahashi K, Saito K: ABO-incompatible kidney transplantation. Transplant Rev (Orlando) 2013;27:1–8. 22902167 10.1016/j.trre.2012.07.003

[17] Klein K, Susal C, Schafer SM, Becker LE, Beimler J, Schwenger V, Zeier M, Schemmer P, her-Goeppinger S, Scherer S, Opelz G, Morath C: Living donor kidney transplantation in patients with donor-specific HLA antibodies enabled by anti-CD20 therapy and peritransplant apheresis. Atheroscler Suppl 2013;14:199–202. 10.1016/j.atherosclerosissup.2012.10.030 23357165

[18] Bentall A, Cornell LD, Gloor JM, Park WD, Gandhi MJ, Winters JL, Chedid MF, Dean PG, Stegall MD: Five-year outcomes in living donor kidney transplants with a positive crossmatch. Am J Transplant 2013;13:76–85. 10.1111/j.1600-6143.2012.04291.x 23072543

[19] Bagnasco SM, Zachary AA, Racusen LC, Arend LJ, Carter-Monroe N, Alachkar N, Nazarian SM, Lonze BE, Montgomery RA, Kraus ES: Time course of pathologic changes in kidney allografts of positive crossmatch HLA-incompatible transplant recipients. Transplantation 27-2-2014;97:440–445. 10.1097/01.TP.0000437177.40551.f4 24531821

[20] Meier-Kriesche HU, Kaplan B: Waiting time on dialysis as the strongest modifiable risk factor for renal transplant outcomes: a paired donor kidney analysis. Transplantation 27-11-2002;74:1377–1381. 12451234 10.1097/00007890-200211270-00005

[21] Montgomery RA, Lonze BE, King KE, Kraus ES, Kucirka LM, Locke JE, Warren DS, Simpkins CE, Dagher NN, Singer AL, Zachary AA, Segev DL: Desensitization in HLA-incompatible kidney recipients and survival. N Engl J Med 28-7-2011;365:318–326. 10.1056/NEJMoa1012376 21793744

[22] Hirsch HH, Steiger J: Polyomavirus BK. Lancet Infect Dis 2003;3:611–623. 14522260 10.1016/s1473-3099(03)00770-9

[23] Hirsch HH, Brennan DC, Drachenberg CB, Ginevri F, Gordon J, Limaye AP, Mihatsch MJ, Nickeleit V, Ramos E, Randhawa P, Shapiro R, Steiger J, Suthanthiran M, Trofe J: Polyomavirus-associated nephropathy in renal transplantation: interdisciplinary analyses and recommendations. Transplantation 27-5-2005;79:1277–1286. 15912088 10.1097/01.tp.0000156165.83160.09

[24] Hirsch HH: BK virus: opportunity makes a pathogen. Clin Infect Dis 1-8-2005;41:354–360. 16007533 10.1086/431488

[25] Fishman JA: BK virus nephropathy—polyomavirus adding insult to injury. N Engl J Med 15-8-2002;347:527–530. 12181409 10.1056/NEJMe020076

[26] Mylonakis E, Goes N, Rubin RH, Cosimi AB, Colvin RB, Fishman JA: BK virus in solid organ transplant recipients: an emerging syndrome. Transplantation 27-11-2001;72:1587–1592. 11726814 10.1097/00007890-200111270-00001

[27] Mengel M, Marwedel M, Radermacher J, Eden G, Schwarz A, Haller H, Kreipe H: Incidence of polyomavirus-nephropathy in renal allografts: influence of modern immunosuppressive drugs. Nephrol Dial Transplant 2003;18:1190–1196. 12748354 10.1093/ndt/gfg072

[28] Binet I, Nickeleit V, Hirsch HH, Prince O, Dalquen P, Gudat F, Mihatsch MJ, Thiel G: Polyomavirus disease under new immunosuppressive drugs: a cause of renal graft dysfunction and graft loss. Transplantation 27-3-1999;67:918–922. 10199744 10.1097/00007890-199903270-00022

[29] Bressollette-Bodin C, Coste-Burel M, Hourmant M, Sebille V, ndre-Garnier E, Imbert-Marcille BM: A prospective longitudinal study of BK virus infection in 104 renal transplant recipients. Am J Transplant 2005;5:1926–1933. 15996241 10.1111/j.1600-6143.2005.00934.x

[30] Barbosa D, Kahwaji J, Puliyanda D, Mirocha J, Reinsmoen N, Lai CH, Villicana R, Peng A, Jordan SC, Vo A, Toyoda M: Polyomavirus BK viremia in kidney transplant recipients after desensitization with IVIG and rituximab. Transplantation 15-4-2014;97:755–761. 10.1097/01.TP.0000437671.78716.f3 24686425

[31] Habicht A, Broker V, Blume C, Lorenzen J, Schiffer M, Richter N, Klempnauer J, Haller H, Lehner F, Schwarz A: Increase of infectious complications in ABO-incompatible kidney transplant recipients—a single centre experience. Nephrol Dial Transplant 2011;26:4124–4131. 10.1093/ndt/gfr215 21622990

[32] Sharif A, Alachkar N, Bagnasco S, Geetha D, Gupta G, Womer K, Arend L, Racusen L, Montgomery R, Kraus E: Incidence and outcomes of BK virus allograft nephropathy among ABO- and HLA-incompatible kidney transplant recipients. Clin J Am Soc Nephrol 2012;7:1320–1327. 10.2215/CJN.00770112 22626962 PMC3408120

